# Unveiling the Efficacy of Physiotherapy Strategies in Alleviating Low Back Pain: A Comprehensive Review of Interventions and Outcomes

**DOI:** 10.7759/cureus.56013

**Published:** 2024-03-12

**Authors:** Priya Tikhile, Deepali S Patil

**Affiliations:** 1 Musculoskeletal Physiotherapy, Ravi Nair Physiotherapy College, Datta Meghe Institute of Higher Education & Research, Wardha, IND

**Keywords:** core strengthening, postural balance, core stability, chronic non-specific low-back pain, low-back pain (lbp), therapeutic intervention

## Abstract

Low back pain (LBP) presents a significant burden globally, affecting individuals of all ages, but it is more common in adults aged 30-60 years old and demographics including race, ethnicity, and socioeconomic status. Physiotherapy interventions are commonly employed to manage LBP due to their non-invasive nature and potential for addressing underlying biomechanical dysfunctions. This comprehensive review aims to evaluate the efficacy of various physiotherapy strategies in alleviating LBP, considering a range of interventions and their associated outcomes. Through a thorough examination of existing literature from January 2017 to October 2023, this review synthesises evidence on the effectiveness of interventions such as manual therapy, exercise therapy, electrotherapy modalities, and education-based approaches. The review also scrutinizes the comparative effectiveness of different physiotherapy modalities and their suitability for specific patient populations, considering factors such as chronicity, severity, and underlying pathology. By critically evaluating the evidence base, this review aims to provide insights into the most effective physiotherapy strategies for alleviating LBP, chronic low back pain (CLBP) and chronic nonspecific low back pain (CNLBP) and guiding clinical practice toward evidence-based interventions.

The Visual Analogue Scale and Numerical Pain Rating Scale for pain, Oswestry Disability Index and Roland-Morris Disability Questionnaire for disability, Modified-Modified Schober Test for measurement of lumbar flexion and extension and static and dynamic balance for assessing postural stability and balance were among the measures used to foresee enhancements in pain, disability, balance, and LBP symptoms. Twenty-one studies that fulfilled the criteria for inclusion (aged 20 to 50 years and of both genders) were added to the review. Exercises for core stability, strengthening, orthosis (a medical device designed to support, align, stabilise, or correct musculoskeletal structures and functions), transcutaneous electrical nerve stimulation, heat massage therapy, interferential current (a form of electrical stimulation used in physical therapy), Mulligan’s mobilization (a manual therapy technique), low-level laser therapy, and McGill stabilization exercises (core exercises) were among the therapeutic strategies.

The McKenzie method (back exercises), ultrasound, sensory-motor training, Swiss ball exercises, and other techniques reduced pain and enhanced strength, balance, and ease of daily activities. Every therapeutic approach has an impact on recovery rates ranging from minimal to maximal. Conventional physical therapy is less effective than most recent advanced techniques like mobilisation and exercises. In summary, the integration of manual techniques, orthoses and alternative intervention strategies with conservative therapeutic approaches can effectively alleviate pain, enhance function and yield better overall outcomes. To get more information about the optimal dosage, therapeutic modalities and long-term effects of these treatments, more admirable research is required. This paper aims to expand the scientific discourse by exploring non-traditional physiotherapy interventions and assessing their efficacy in light of the rigorous standards set forth by the latest WHO guidelines.

## Introduction and background

Most individuals likely have back pain at any stage in their life, which is a well-known and widespread health issue. However, it is often ignored. The most common reason for activity limitation is low back pain (LBP) [1]. Given the lack of global consensus regarding the description of chronic low back pain (CLBP), it was described as "pain and discomfort, localizing below the costal margin and above the inferior gluteal folds, regardless of referred leg pain enduring for at least 12 weeks". The World Health Organization's latest guidelines for the management of CLBP have set a new benchmark in evidence-based care, providing a comprehensive review of effective treatment modalities [2]. Many factors contribute to CLBP, such as biopsychosocial factors like genetic predisposition, age, sex, body weight, stress, anxiety, occupation, and lifestyle factors. CLBP is linked to trunk muscle weakness, especially in the deep trunk muscles, along with issues with coordination and trunk proprioception. This may increase the risk of lumbar spine instability, additional spine injuries, and eventually, a reduction in physical activity [3]. Muscle atrophy and limited movement occur when back pain worsens over several months. Chronic conditions result in a reduction in the muscle's cross-section region surrounding the vertebral bodies, which deteriorates back pain and causes additional injury and recurrence [4]. As per the World Health Organization, CLBP is a common and costly condition, affecting an estimated 1 in 6 adults globally. However, most people with CLBP can recover well with non-surgical management in primary and community care settings [5].

Changes in the lumbar multifidus muscle activation sequence, delayed recruitment within the transverse abdominis muscle, and poor efficiency have all been characterised in patients with CLBP. Postural and balance problems can also result from abnormalities in the impulse transported by mechanoreceptors and dysfunction within the peripheral proprioceptive system of paraspinal muscles. Previous studies have suggested that impaired postural control may result from a decrease in lower back muscle coordination and an increase in active muscle tension [6]. LBP can be treated in several manners such as medication, therapeutic exercises and rest. By retraining and building muscle endurance, these exercises aim to preserve and stabilise the proper position of the muscles. Additionally, they attempt to stabilise the spinal cord in an effort to lessen pain and enhance function. Activities carried out by an individual to deal with their balance limitations are included in static and dynamic balance training [7].

Pain is conveyed by nociceptors, which are specialised peripheral sensory neurons. The impulses from the skin are then transmitted to higher brain centres in the form of electrical impulses warning of possibly harmful stimuli. The neuronal body of nociceptors, which are pseudo-unipolar primary somatosensory neurons, is found in the dorsal root ganglion. These axons are bifurcated, with the central branch synapsing on second-order neurons in the dorsal horn of the spinal cord and the peripheral branch innervating the skin. Second-order neurons direct the sensory-discriminative and affective-cognitive characteristics of pain, correspondingly by projecting to the mesencephalon and thalamus which subsequently link with somatosensory and anterior cingulate cortices. Peripheral and central sensitization processes may take place if the unpleasant stimulus continues, transforming acute pain into chronic pain [8].

An operational definition of central sensitization is an increase in neural signalling in the central nervous system (CNS) that causes hypersensitivity to pain [9]. Many chronic pain conditions, including fibromyalgia, headaches, osteoarthritis, temporomandibular disorders, LBP and lateral epicondylalgia, can cause central sensitization. Even with growing understanding of the mechanisms causing central sensitization, treatment still needs to be improved. Both central and peripheral sensitization plays a crucial part in LBP cornification. As a matter of fact, even slight alterations in posture have the potential to cause chronic inflammation in the muscles, ligaments and joints that support the low back, thereby exacerbating the condition of peripheral and central sensitization. Additionally, the rich innervation of joints, discs and bones is provided by A-delta fibres whose constant stimulation may readily lead to central sensitization [8].

Most people with backache would have likely experienced acute episodes of LBP at some point in their lives. LBP can be a dull ache or sharp pain. It can also cause pain to radiate into other areas of the body, especially the legs. LBP can restrict a person’s movement, which can affect their work, education and community engagement. It can also cause problems with sleep, mood and distress. In most cases of acute LBP, symptoms go away on their own and most people will recover well. However, for some people, the symptoms will continue and turn into chronic pain. People with LBP may also experience spine-related leg pain (referred as sciatica or radiating pain). This is often described as a dull pain or a sharp, electric shock-like tingling. Numbness or tingling and weakness in some muscles may be experienced with the leg pain. When associated with LBP, radicular signs and symptoms, previously mentioned, are often due to the involvement of a spinal nerve root. Some people may experience radicular symptoms without LBP when a nerve is compressed or injured distal to the spinal column [10].

There were 28 episodes of LBP for every 1000 people annually. A yearly occurrence of sciatica-related LBP was reported to be 11.6/1000. Men have greater events of LBP (32.0) in comparison with females (23.2). The age group between 25 and 64 has the highest incidence [11]. In 1990, the age-standardised point prevalence of LBP was 8.20% worldwide; by 2017, it had dropped to 7.50%. The majority was higher in females compared to males. This was 8.86% for females in 1990 and 8.01% for males in 2017, with 7.47% for males in 1990 and 6.94% for males in 2017. The estimated prevalence of LBP was 377.5 million in 1990; however, because of the significant global population growth between 1990 and 2017, the number rose to 577 million. The incidence of LBP rose as individuals ages, peaking around the ages of 80 and 89, after which it somewhat declined. Between 1990 and 2017, this trend was seen in both males and females [12].

In every age group, the spine was the primary cause of LBP. But as people get older, the possible sources of LBP - such as the hip with the spine and the hip with the sacroiliac joint tend to get combined. Therefore, when treating middle-aged and older patients, doctors should always consider additional causes of LBP [13]. Whenever a patient exhibits signs or symptoms that increase the probability of a serious pathology, further investigations beyond the scope of the clinical examination are necessary. Blood tests, nerve conduction tests, imaging techniques like computed tomography, computed radiography, magnetic resonance imaging, dual-energy X-ray absorptiometry, and infrequently myelography are pertinent investigations. Patients with LBP rarely need surgical procedures since it is seldom a symptom of severe pathology [14].

Since the LBP is a frequent condition. Linked to high rates of healthcare requirements and absenteeism from work, prevention is crucial to minimizing the severe suffering and hefty associated expenses. The disparity between primary and secondary prevention is frequently emphasized. The goal of primary prevention is typically to stop a disease from developing in healthy individuals, whereas the purpose of secondary prevention is to prevent the recurrence of the disorder. Because lumbar support belts support the trunk and prevent pain-producing events from occurring due to over-flexion, they may help prevent LBP. Exercise may assist in avoiding LBP because it improves trunk flexibility, strengthens the back muscles and increases blood flow to the spine, muscles and intervertebral discs, which reduces injury and speeds up healing. Workplace health hazards include being subjected to vibration, carrying loads, vigorous labour, a fixed work posture, repeated bends and twirling which are the focus of ergonomic interventions [11].

According to reports, postural control adapts to changes in involvements from high-threshold nociceptive muscle afferents. CLBP has been associated with anomalies in the morphology and initiation of the lumbar multifidus and abdominal muscles. Reduced multifidus geometry has been associated with worse functional disability indices and increased pain in people with chronic non-specific mechanical LBP. Furthermore, there was a correlation between poorer dynamic balance and decreased thickness of the abdominal musculature.

This review article's main goal is to perform a thorough evaluation of the possible advantages provided to people with LBP by both newly developed modalities and conventional interventional strategies. While adhering to the stringent evidence requirements of the WHO guidelines, this review will provide a critical analysis of non-traditional physiotherapy techniques, aiming to broaden the array of evidence-based interventions available for CLBP management. The purpose of this review is to evaluate the efficacy of various physiotherapy strategies in alleviating LBP, considering a range of interventions and their associated outcomes. By critically evaluating the evidence base, this review aims to provide insights into the most effective physiotherapy strategies for alleviating LBP, CLBP and CNLBP and guiding clinical practice towards evidence-based interventions.

## Review

Methodology

*Data Sources, Search Engines and *Eligibility Criteria

**Figure 1:**
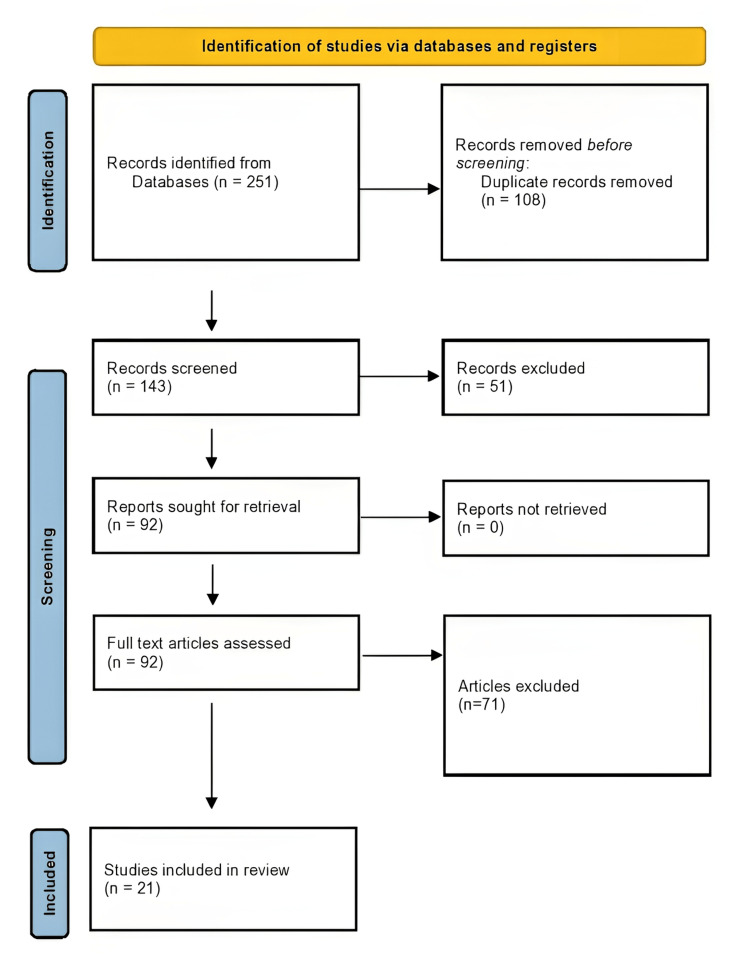
Preferred Reporting items for Systematic Reviews and Meta-Analyses flowchart

A database search was conducted on Cochrane Library, PEDro, PubMed and Google Scholar for randomized and non-randomized clinical trials in the English language to assess the effect of different physiotherapeutic treatments on regaining LBP symptoms in patients. The study includes younger and middle-aged adults with non-specific CLBP that have persisted for more than three months. This type of LBP is known as non-specific mechanical LBP, meaning it lacks a clear, identifiable specific anatomical or neurophysiological causing factor. Using the combination of the keywords non-specific CLBP, core stability exercises, strengthening, and advanced physiotherapy, 251 articles were found between January 2017 and October 2023. A total of 21 of the 251 articles were suitable for review. An overview of the literature selected in compliance with the Preferred Reporting Items for Systematic Reviews and Meta-Analyses (PRISMA) guidelines can be found in Figure 1.

Summary of the articles that were reviewed is mentioned in Table 1.

**Table 1:** Summary of the articles reviewed RCT: randomized controlled trial; CLBP: chronic low back pain;  VAS: visual analogue scale; MODQ: modified oswestry disability questionnaire; RS: rhythmic stabilisation; LBP: low back pain; CLBP: chronic low back pain; CNSLBP: chronic nonspecific low back pain; MMST: modified-modified schober test; ODI: ODI: oswestry disability index; TENS: transcutaneous electrical nerve stimulation; NPS: numerical pain scale;  MPQ: McGill pain questionnaire; PPT: pain pressure threshold; CI: interferential current; CA: aussie current; IC: interferential current; PG: placebo group; NSCLBP: non-specific chronic low back pain; LROM: lumbar range of motion; CSE: core stabilization exercises; KT: kinesiology taping; PNRS: pain numeric rating scale; RMDQ: Roland-Morris disability questionnaire; SF-MPQ: short form - McGill pain questionnaire; MFI-20: multidimensional fatigue inventory; BDI: beck depression inventory; sEMG: surface electromyography; SSR: sympathetic skin response; HMG: heat massage group; PTG: physical therapy group; % MVC LM: percentage of the maximum voluntary contractions of lumbar multifidus; PNF: proprioceptive neuromuscular facilitation; LM: lumbar multifidus; TrA: transverse abdominis; MF: lumbar multifidus; PPCE: progressive postural control exercise; NPRS: numerical pain rating scale; TSK: tampa scale for kinesiophobia; , ROM: range of motion; QOL; quality of life; UST: ultrasound therapy; KT: kinesio taping; SMT: sensory motor training; LLLT: low-level laser therapy; SNAGs: sustained natural apophyseal glides; LSO: lumbosacral orthoses; IFT: interferential therapy.

Sr. No	Authors and year	Study type	Outcome measures	Intervention	Results	Conclusions	Inference
1	Singh et al. 2023 [15]	RCT (n=60 individuals with CLBP)	VAS, MODQ	Group A: Rhythmic stabilisation Group B: Back strengthening exercises	LBP significantly p<0.001 enhanced with the experimental group, and the MODQ considerably reduced, while the control group showed no significant changes	RS improves LBP and disability, with the experimental group showing greater improvement than the control group in the program.	In the rehabilitation of CLBP and accompanying disability, physiotherapy approaches that include RS may be more beneficial than those that only use conventional treatments which include Stretches and core strengthening exercises to improve flexibility and core stability. Cat-camel stretch, single knee-to-chest stretches, abdominal curl-ups, and abdominal bridging followed by unilateral straight leg lowering for both legs.
2	Abdelhaleem et. al 2023 [16]	RCT (n=60 individuals with CNSLBP)	VAS, MMST, ODI	Group A: Traditional physical therapy. Group B: Conventional physiotherapy in addition to ankle stability exercises	Group B showed a substantial reduction of ODI and VAS post-treatment compared to Group A (p < 0.05) and a significant increase of MMST (p < 0.05).	Ankle stability exercises enhanced ODI, MMST, and pain when added to conventional therapy exercises: Hot pack, TENS, stretching exercises for the lower back and hamstrings, clamshell exercise for activation of gluteus medius muscles, and core strengthening.	Enhancing results for people with persistent LBP with the inclusion of ankle stability exercises in physical therapy interventions highlights the possibility for a holistic approach aimed at reducing pain, improving disability and improving flexibility.
3	Paula et al. 2023 [17]	RCT (n=125 individuals with CLBP)	NPS, MPQ and PPT	5 groups: CI4kHz/100Hz, CI4kHz/2Hz, CA4kHz/100Hz, CA4kHz/2Hz and placebo. For thirty minutes, each participant underwent a single session with either Aussie current or interferential current.	Significant differences in NPS and MPQ groups (p<0.05) for IC 4 kHz/100 Hz and IC 4 kHz/2 Hz compared to PG.	Interferential current, regardless of modulation had immediate analgesic effects in CLBP individuals, superior to Aussie current.	The results indicate that for participants with CLBP, IC performed better compared to Aussie Current in terms of giving rapid reduction in pain, regardless of modulation. This emphasises how effective Interferential Current may be as a pain management technique for CLBP sufferers.
4	Ogunniran et al. 2023 [18]	RCT (n=35 NSCLBP)	Pain intensity, functional disability, psychological state, sleep disturbance, kinesiophobia and LROM had been evaluated.	Three groups: Kinesio Taping + Core Stability Exercise, CSE and KT only.	Pain reduction, improved function, better psychological state, reduced kinesiophobia, reduced disruption to sleep and improved LROM in all groups post-intervention.	All participants improved, but the KT + CSE group had superior clinical outcomes	When Kinesio Taping and Core Stability Exercise were combined, all participants to those having NSCLBP improved more significantly and comprehensively.
5	Kim et al. 2023 [19]	Randomized Controlled Feasibility Trial (n=40 LBP participants)	PNRS, ODI, RMDQ, SF-MPQ, MFI-20, BDI, sEMG, SSR	HMG group: simultaneous heat massage therapy using a mechanical device PTG: conventional physical therapy	In both groups, there was no significant improvement in PNRS, ODI, RMDQ or SF-MPQ. Prior to PTG, BDI in HMG improved. MFI-20 has improved, yielding superior HMG outcomes.	It was demonstrated that both interventions were successful in reducing LBP and pain-related impairment.	When compared to traditional physical treatment the benefits of simultaneous heat massage therapy are particularly noticeable in the alleviation of fatigue (MFI-20) and earlier improvement in depression (BDI).
6	Baig et al. 2022 [20]	RCT (150 individuals with CLBP)	VAS, MMST, ODI, sEMG, % MVC LM	Intervention group: bilateral asymmetrical limb PNF, comparison group: Swiss ball exercises	Pain, ODI disability, and % MVC LM significantly improved (P < .001) in the PNF group compared to the comparison group.	Bilateral asymmetrical limb PNF exercises improved pain, disability, and LM activity in CLBP patients more than Swiss ball exercises.	PNF exercises were beneficial than standard Swiss ball exercises CLBP.
7	Wang et al. 2022 [21]	RCT (n=34 individuals with CLBP)	VAS, ODI, RMDQ, contractility of TrA and MF, and the capacity to regulate an unchanged posture	Exercise groups for core stability and progressive postural control group	VAS, ODI, and RMDQ scores significantly decreased in both groups. Percentage change in TrA and left MF thickness increased and the sway area of the centre of pressure during static stance with eyes open decreased in both groups.	For individuals with CLBP, PPCE has advantages equivalent to those of core stability exercises.	Exercises for core stability and progressive postural control have comparable effective benefits. These advantages cover all outcomes. The adaptability of these therapy modalities which gives medical professionals choices when creating successful rehabilitation plans for CLBP patients.
8	Aguilar‑Ferrándiz et al. 2022 [22]	RCT (n=58 individuals with NSCLBP)	RMDQ, ODI, Tampa Scale for Kinesiophobia, Pittsburgh Sleep Quality Index, NPRS	Kinesio taping group and Transcutaneous electrical nerve stimulation group	Statistically significant group differences in NPRS, ODI, TSK	The analgesic current, kinesio taping and exercise therapy when combined together relieved pain, disability, anxiety, depression and improved sleep patterns.	Exercises, kinesio taping and analgesic current when incorporated together yielded favourable results.
9	Rodríguez-Huguet et al. 2022 [23]	A single blind RCT (n=50 individuals with NSCLBP)	Pain, PPT, ROM, functionality and QOL	Vacuum treatment combined with a group for core therapeutic exercises and a group for a physical therapy: supine bridge, prone bridge, side bridge, dead bug and bird dog	Substantial variations were observed right after treatment in the physical therapy group.	Myofascial vacuum therapy intervention improved pain, mobility, pressure pain threshold, functionality and quality of life.	Though each intervention showed potential, myofascial vacuum therapy, in conjunction with core, therapeutic exercises can be thought of as a comprehensive, effective method for managing pain and improving multiple functional domains QOL in patients with persistent LBP that is not specific.
10	Fouda et al. 2021 [24]	RCT (n=60 individuals with CLBP)	Trunk endurance, spinal mobility, functional impairment	Group A: conventional physical therapy + Rhythmic stabilisation training. Group B: traditional physical therapy + integrating of isotonic techniques. Group C: An integration of several isotonic workouts and training in rhythmic stabilisation.	Following treatment, there were significant differences (p < 0.05) in the outcomes evaluated across the groups.	Rhythmic stabilisation training combined with isotonic exercises for PNF was better in treating the patient than any strategy by itself.	When combating NSCLBP, an integrated approach that combines stability training and isotonic exercises may be more effective than utilising each technique separately.
11	Hlaing et al. 2021 [25]	RCT (n=36 individuals with NSCLBP)	Proprioception, standing balance, muscle thickness of TrA and LM, VAS, MODQ, Tampa Scale for Kinesiophobia	Core stabilisation exercise group and strengthening exercise group	Compared to the group that performed strengthening exercises, the CSE group showed considerably greater progress	Core stabilisation exercise is superior to enhancing exercise	The group that performed core stability exercises showed much superior results than the group that performed strengthening activities, as determined by a thorough assessment of multiple parameters, such as pain, disability, muscle thickness, and kinesiophobia
12	Otadi et al. 2021 [26]	A randomised clinical trial (n=24 individuals with NSCLBP)	Static stability, dynamic balance, pain and function	The interventional group, TENS combined with diaphragm training and the control group: TENS alone	The intervention group showed higher improvements in pain, static strength and dynamic balance than the control group. Both groups saw an improvement in function	Compared to TENS alone, diaphragm training plus TENS resulted in larger gains in pain, static strength, and balance	The fact that all groups' functions were improved suggests that the therapies were successful; nonetheless, the experimental group performed better in two important domains: pain and physical stability
13	Sipko et al. 2021 [27]	A randomised control trial (n=53 individuals with CLBP)	Centre of pressure, tandem and one-leg standing tests	The intervention group, PNF and control group	In the leg stand test mediolateral plane, pain and sample entropy declined quickly following the exercise and either reached or even surpassed the baseline values	For patients with CLBP, a single PNF exercise session may be helpful for pain management and regulating posture	This indicates that PNF exercises could be a viable strategy for CLBP patients to enhance their postural stability and manage their pain
14	Nugraha et al. 2021 [28]	RCT (n=20 individuals with NSCLBP	NRS, Goniometer, ODI	Control group: UST + KT + PNF Experimental group: UST + KT + sensory motor training	The outcomes demonstrated that each group's low back disability, ROM and pain had improved	When it comes to enhancing pain, ROM and disability, the UST + PNF + KT combined performs just as well as the UST+SMT+KT together	The research's main conclusion showed that each of the treatment and control groups had improvements in low back impairment, ROM and disability
15	Seo et al. 2020 [29]	RCT (49 participants with CLBP)	VAS, MMST, RMDQ	SNAGs with the LLLT group, the SNAGs group and the control group were segregated into three groups	Following treatment in the SNAGs with the LLLT group and the SNAGs group, the VAS and MMST scores increased considerably. Following the treatment program, the RMDQ score of both the control group and the SNAGs with LLLT SNAGs increased substantially	Combining LLLT with Mulligan’s mobilization therapy is a beneficial way to enhance ROM and function while lowering pain	For those with CLBP, Mulligan’s mobilization in addition to LLLT is an efficient way to decrease pain and enhance ROM and function
16	Abass et al. 2020 [30]	RCT (n=40 participants with NSCLBP)	VAS, ODI, Tampa Scale of Kinesiophobia questionnaire, back muscle endurance	Experimental group: Lumbar stabilisation + conventional therapy Control groups: Traditional therapy	There was a significant reduction in pain intensity disability and an increase in back muscle endurance	Augmenting conventional physiotherapy with lumbar stabilisation exercises achieved a better reduction in disability than conventional therapy alone	For individuals with NSLBP, adding lumbar stability exercises to traditional therapy produced noteworthy improvements
17	Anggiat et al. 2020 [31]	Quasi-experimental study (n=36 participants with NSCLBP)	ODI	Three groups: PNF, McKenzie and control group	PNF demonstrated that its impact on the functional disability score is greater than that of the McKenzie technique	Functional disability on NSLBP changed with the implementation of the PNF and McKenzie method, improving functional disability compared to McKenzie method	In terms of disability, the PNF approach outperformed the McKenzie approach. This demonstrates how PNF may be useful in treating functional impairment brought on by NSLBP
18	Azadinia et al. 2019 [32]	RCT (n=44 participants with LBP)	At three different levels of difficulties for postural tasks - eyes open on a rigid surface, eyes closed on a rigid surface and eyes closed on a foam surface -the centre of pressure fluctuations were measured during standing	Intervention group: LSO in addition to conventional physiotherapy and only conventional physiotherapy group	LSO and conventional physical therapy techniques were used as an intervention, although in people experiencing LBP, this did not affect the temporal structures of postural sways	Rehabilitation approaches that only address the correction of peripheral mechanics, such as LSO or conventional physiotherapy modalities, are unable to modify the way the postural control system behaves	Both LSO and conventional physical therapy methods were effective in helping LBP patients manage their postural sways
19	Kotteeswaran et al. 2019 [33]	RCT (n=60 participants with LBP)	ODI	Group A: Mulligan’s technique with IFT and Group B: conventional physiotherapy abdominal strengthening exercise with IFT	ODI values in Group B disability levels were much higher than those of Group A	Better improvement in reducing LBP in Mulligan’s technique than traditional abdominal strengthening exercise	When it comes to lowering LBP related impairment, the Mulligan’s technique works better than conventional physiotherapy methods
20	Malla et al. 2018 [34]	RCT (n=30 participants with NSLBP)	VAS, digital inclinometer, RMDQ	Group A: Swiss ball exercises & Group B: PNF exercises	Considerable improvements in both approaches	Using a Swiss ball exercise for motor control and rhythmic stabilization approach, patients with NSLBP demonstrated substantial improvements in their ROM, pain, and disability	This suggests that both strategies may be useful in the management of NSLBP, providing medical professionals with a multitude of choices for creating rehabilitation plans tailored to the specific requirements of each patient
21	Farajzadeh et al. 2017 [35]	RCT (n=30 participants with NSLBP)	VAS, Quebec LBP Disability Scale questionnaire, inclinometer and Biodex Balance System	McGill stabilization exercises group and conventional physiotherapy group	Comparing each of the groups, there were no appreciable variations in pain, disability, and ROM. Notably, among the dynamic postural stability variables, changes were observed	In order to sustain balance during everyday tasks like walking, McGill stabilisation exercises might enhance improved dynamic postural balance variables	Based on this, it appears that although there are some similarities between the two methods, McGill stabilizing exercises could be a more useful intervention for enhancing dynamic postural balance, especially when it comes to regular walking

Discussion

LBP is a common health issue that has a significant negative impact on a person's personal life as well as their ability to perform in community roles. The specific cause of LBP in many cases remains elusive, leading to a classification of nonspecific LBP for these patients. It has been suggested that chronic nonspecific low back pain may be exacerbated by impaired or inadequate motor control in the deep trunk musculature. Individuals with chronic nonspecific low back pain frequently experience changes like delayed transverse abdominis activation, affected lumbar multifidus recruiting patterns, endurance limitations and reduced repositioning accuracy. Postural and balance problems can also result from abnormalities in the data conveyed by mechanoreceptors and deficits in the peripheral proprioceptive system of paraspinal muscles. A reduction in lower back muscle coordination and an increase in active muscle tension may be the cause of postural control impairment.

In recent years, we have seen a widespread increase in core stability, characterised by a reduction in the tonic activity of the transversus abdominus during gait and extremity activity, as well as a reduced action of the lumbar multifidi and transversus abdominus. Reduction in the stability provided by the lumbar spine and a rise in the strain and stress on its joints and ligaments can result from the malfunction of these muscles. There are several health advantages to the exercise therapy approach. The majority of physical therapists in the area typically treat CLBP with physiotherapy. It includes stretching, strengthening exercises and some targeted exercises for the home plan in addition to electrotherapy modalities (ultrasound, TENS, interferential therapy, etc.). Physical therapies use ultrasound, LLLT, heat and cold therapy, and IFT to treat pain [36]. LBP is often associated with altered muscle function, including delayed activation, abnormal recruitment patterns, and endurance limitations. These changes can contribute to postural dysfunction and impaired balance, ultimately impacting daily activities and potentially worsening pain. Physiotherapy offers various interventions to address these issues. Stability exercises, for instance, aim to restore normal movement patterns by promoting coordinated contraction of core muscles. Additionally, sensory-motor training utilizes exercises like balance training on uneven surfaces to stimulate the proprioceptive system, helping to normalize muscle response patterns and improve postural awareness and control. By addressing both muscle dysfunction and sensory deficits through targeted physiotherapy interventions, individuals with LBP can experience improved pain management, enhanced functional movement, and increased overall well-being.

The inclusion of spinal manipulation within the treatment paradigm for CLBP aligns with the latest recommendations from the WHO. The WHO now endorses spinal manipulation as an effective intervention for managing nonspecific CLBP, positioning it as a pivotal aspect of comprehensive care [37]. For those with CLBP, this manual therapy technique which applies regulated force to the vertebral joints has shown to provide pain relief and improved functional outcomes [38]. The proposed mechanism of action for spinal manipulation includes neurophysiological effects that modulate pain and the restoration of normal joint kinematics [39]. Research indicates that the integration of spinal manipulation into a CLBP management plan, when performed by trained professionals, can enhance patient outcomes by addressing the multifaceted etiology of the condition, including biomechanical and neuromuscular dysfunction [37]. By decreasing skin resistance and activating sensory nerve fibres, IFT enables currents to reach deeper tissues. This treatment not only lessens pain but also improves blood circulation, muscle relaxation and proprioceptive sensibility, all of which contribute to an improvement in balance. The PNF method improves the proprioception of the lumbar muscles and permits training in sensory-motor control [40]. According to a study by Fouda et al. neuromuscular control through stimulation of muscle and joint proprioceptors as well as sensory inputs from peripheral organs, affects the CNS motor outputs and encourages functional activity of daily living. The authors recommended that patients with CLBP be treated with a combination of isotonic exercises and the rhythmic stabilisation training technique of PNF [24]. Sipko et al. evaluated how PNF affected people with persistent LBP on the tandem, one-leg standing and centre of pressure tests. They showed that PNF exercise, even for just one session, might help CLBP patients with their pain and postural control [27]. Mavromoustakos et al. examined the impact of a six-week program of general exercise versus PNF on pain and disability in patients with chronic low back pain. The results showed that pain was reduced higher in the PNF group compared to the general exercise category, and it was suggested that structured programs implementing all PNF techniques could be utilised to treat CLBP [40].

We methodically gathered all of the information that was available and evaluated the possibility of bias in pertinent trials and research for this review. Out of 251 studies, 21 had homogeneous populations participant demographics (age range and gender), the type of low back pain (chronic, acute, nonspecific), and the intervention protocols employed and designs, enabling comparisons. This validates our conclusions drawn from the finest available data. However, it is critical to recognise the inherent limits of the information provided. In order to reach more firm findings, more researches are required. Therefore, more extensive clinical research with larger sample sizes is necessary for a thorough assessment of these results.

## Conclusions

The scrutiny of studies shows how treatment approaches are successful in reducing pain, promoting better function and enhancing patient satisfaction. Rhythmic stabilisation is superior to traditional physiotherapy in terms of reducing pain and minimising disability. Core stability exercises outperform a variety of modalities, including kinesio taping, heat massage therapy, postural control exercises, sensory-motor training, McGill stabilisation exercises and lumbar stabilisation. PNF has been demonstrated to enhance balance, pain, postural control and disability. TENS, IFT and UST provide excellent pain relief results. Both LLLT and Mulligan’s mobilisation efficiently lessen pain while enhancing function and ROM. Mulligan’s methods outperform conventional exercises. Treatment outcomes are greatly improved by a combined strategy that includes both standard conventional therapies and advanced treatments.
